# Association of Estimated Long-term Exposure to Air Pollution and Traffic Proximity With a Marker for Coronary Atherosclerosis in a Nationwide Study in China

**DOI:** 10.1001/jamanetworkopen.2019.6553

**Published:** 2019-06-28

**Authors:** Meng Wang, Zhi-Hui Hou, Hao Xu, Yang Liu, Matthew J. Budoff, Adam A. Szpiro, Joel D. Kaufman, Sverre Vedal, Bin Lu

**Affiliations:** 1 Department of Epidemiology and Environmental Health, School of Public Health and Health Professions, University at Buffalo, Buffalo, New York; 2 RENEW Institute, University at Buffalo, Buffalo, New York; 3 Department of Environmental and Occupational Health Sciences, School of Public Health, University of Washington, Seattle; 4 Department of Radiology, Fuwai Hospital, National Center for Cardiovascular Diseases, Chinese Academy of Medical Sciences, Beijing, China; 5 Department of Earth System Science, Tsinghua University, Beijing, China; 6 Department of Environmental Health, Rollins School of Public Health, Emory University, Atlanta, Georgia; 7 Department of Medicine, Division of Cardiology, Harbor UCLA Medical Center, Torrance, California; 8 Department of Biostatistics, University of Washington, Seattle

## Abstract

**Importance:**

Epidemiologic evidence of the mechanisms of the association between long-term exposure to air pollution and coronary heart disease (CHD) is limited and relies heavily on studies performed in Europe and the United States, where air pollution levels are relatively low. In particular, the association between air pollution and CHD in patients with underlying risks for CHD is understudied.

**Objective:**

To determine whether air pollution and proximity to traffic are associated with the coronary artery calcium (CAC) score, a key atherosclerotic marker.

**Design, Setting, and Participants:**

In this prospective, population-based cross-sectional study in a large-scale setting in China, 8867 consecutive patients aged 25 to 92 years with suspected CHD were recruited between November 17, 2015, and September 13, 2017. Participants were excluded if they had previous myocardial infarction, stenting, or coronary artery bypass grafting or incomplete risk factors and exposure data. Each participant underwent assessment of CAC and CHD risk factors at baseline. Data were analyzed from December 2017 to November 2018.

**Exposures:**

Annual means of fine particulate matter with aerodynamic diameter less than 2.5 μm (PM_2.5_), nitrogen dioxide (NO_2_), and ozone (O_3_) were estimated at the participants’ residences using a validated geostatistical prediction model. Exposure to a nearby roadway was also estimated.

**Main Outcomes and Measures:**

Computed tomography measurement of CAC score.

**Results:**

The mean (SD) age of the 8867 participants was 56.9 (10.4) years; 4378 (53.6%) were men. Annual mean (SD) PM_2.5_, NO_2_, and O_3_ measurements were 70.1 (20.0), 41.4 (14.7), and 93.9 (10.5) μg/m^3^, respectively. The mean (SD) CAC score was 91.4 (322.2) Agatston units. Exposure to PM_2.5_ and NO_2_, adjusting for CHD risk factors and multiple pollutants, were independently associated with increases in CAC scores of 27.2% (95% CI, 10.8% to 46.1%) per 30 μg/m^3^ PM_2.5_ and 24.5% (95% CI, 3.6% to 49.7%) per 20 μg/m^3^ NO_2_. For PM_2.5_, odds of both detectable CAC (Agatston score >0; odds ratio, 1.28; 95% CI, 1.13 to 1.45) and severe CAC (Agatston score >400; odds ratio, 1.59; 95% CI, 1.20 to 2.12) were increased. Associations of CAC with PM_2.5_ and NO_2_ were greater among male participants (PM_2.5_: 42.2%; 95% CI, 24.3% to 62.7%; NO_2_: 45.7%; 95% CI, 25.3% to 69.5%) and elderly participants (PM_2.5_: 50.1%; 95% CI, 28.8% to 75.0%; NO_2_: 55.5%; 95% CI, 31.8% to 83.6%) and those with diabetes (PM_2.5_: 62.2%; 95% CI, 30.9% to 101.0%; NO_2_: 31.2%; 95% CI, 13.9% to 51.0%). Independent association with CAC score was 9.0% (95% CI, −1.4% to 20.4%) for O_3_ per 15 μg/m^3^ and 2.4% (95% CI, −0.6% to 5.4%) for distance near roadway per 50% decrease.

**Conclusions and Relevance:**

In this large Chinese study, long-term exposures to PM_2.5_ and NO_2_ were independently associated with severity of CAC. This finding may provide support for the pathophysiological role of coronary atherosclerosis through which air pollution exposure may be associated with CHD.

## Key Points

**Question:**

Are long-term exposure to ambient air pollution and proximity to traffic associated with subclinical atherosclerosis?

**Findings:**

In this population-based cross-sectional study of 8867 Chinese participants, long-term exposure to ambient nitrogen dioxide and fine particulate matter with aerodynamic diameter less than 2.5 μm was independently associated with a higher coronary artery calcium score, a key atherosclerotic marker. Associations with ozone and proximity to traffic were less consistent.

**Meaning:**

Long-term exposure to ambient air pollution may be an important risk factor for coronary atherosclerosis.

## Teaser

This population-based cross-sectional study of 8867 Chinese participants examines the association of long-term exposure to air pollution and proximity to traffic with the coronary artery calcium score, a marker for coronary atherosclerosis.

## Introduction

Ambient air pollution is a major contributor to the global burden of disease, accounting for an estimated 4.2 million deaths in 2015.^1^ Although the effect of air pollution on public health is potentially very large, the current air pollution guidelines used worldwide have relied principally on findings from studies conducted in Europe and North America where air pollution levels are relatively low compared with other areas experiencing more recent rapid industrialization.^2^ Information on associations between exposure and disease from regions with exceptionally high ambient air pollution, such as China, would be useful to produce more accurate estimates of air pollution–attributable disease burden, which in turn can be used to prioritize public health responses, especially in developing countries.

Cardiovascular disease is the leading cause of death worldwide, with available evidence showing that it is associated with long-term exposure to air pollution and proximity to traffic.^3^ A likely pathway underlying these associations involves the initiation or acceleration of atherosclerosis.^3^ Atherosclerosis is a lifelong process; therefore, the effects of air pollution exposure on atherosclerosis are likely to be long term. If an association between air pollution and subclinical atherosclerosis were established, it could provide an opportunity to intervene before disease is manifested clinically by way of community-level efforts to control pollution exposures. Common approaches to detecting subclinical atherosclerosis include noninvasive measurement of coronary artery calcium (CAC) or carotid intima-media thickness.^4^ Compared with carotid intima-media thickness, CAC reflects a more advanced stage of atherosclerosis involving calcification of plaque and is a better predictor of cardiovascular disease.^4^ Associations between air pollution and carotid intima-media thickness have been reported in some previous studies.^5,6,7,8^ Evidence regarding the association between air pollution and coronary atherosclerosis, determined using CAC scoring, is still very limited.^8,9^

China is the most populated country in the world and has a large estimated disease burden associated with air pollution.^1^ Cohort studies of air pollution and cardiovascular health are still scarce and have been largely limited to the investigation of effects on mortality.^10^ Given the increasing public concern regarding air pollution, the Chinese government has recently implemented an unprecedented effort to characterize major air pollutant exposures with more than 1400 widespread monitoring stations established nationwide.^11^ Here we use this valuable resource by applying advanced exposure estimation methods for health effect investigation. We hypothesize that long-term exposure to air pollution or proximity to traffic is associated with increased risk of CAC in a well-characterized cohort of Chinese adults.

## Methods

### Study Population

**Figure 1.**
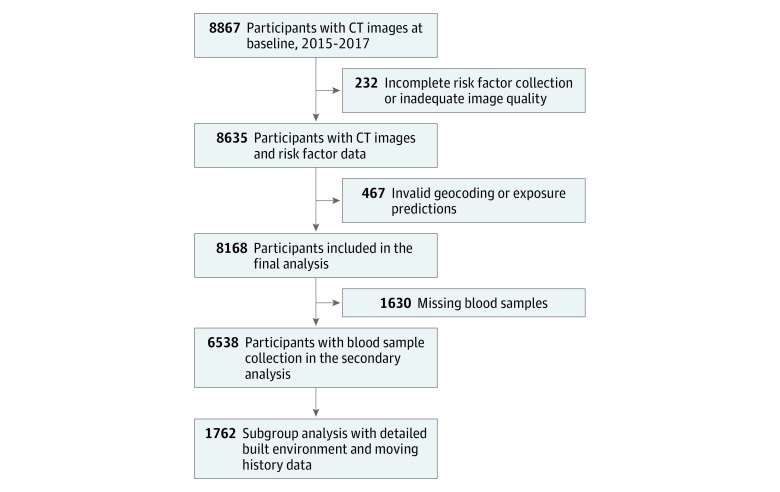
Study Design and Population Flowchart shows study cohort enrollment criteria. CT indicates computed tomography.

This cross-sectional study used baseline data collected from a prospective cohort (the CREATION cohort) of 8867 consecutive patients aged 25 to 92 years at Fuwai Hospital in Beijing, China (eMethods in the Supplement). The participants were suspected of having coronary heart disease (CHD) and for this reason underwent cardiac computed tomography to evaluate the presence and amount of CAC between November 17, 2015, and September 13, 2017. Cardiac imaging was ordered by primary cardiologists. The participants had relatively low pretest probability of CHD (mean [SD], 27.3% [15.1%]); 2587 patients had no symptoms, 5721 had nonanginal chest pain, 449 had atypical angina, and 110 had typical angina. Participants were excluded if they had previous myocardial infarction, stenting, or coronary artery bypass grafting or incomplete risk factors and exposure data. More details regarding the study cohort are available in Figure 1 and eMethods in the Supplement. All participants provided written informed consent, and the study was approved by the institutional review board of the Chinese Academy of Medical Sciences Fuwai Hospital. This report follows the Strengthening the Reporting of Observational Studies in Epidemiology (STROBE) reporting guideline.

### CAC Measurement

Details of the measurement of CAC have been published elsewhere.^12^ In brief, all patients underwent imaging with a second-generation dual-source computed tomography system (Somatom Definition Flash; Siemens Healthcare) with a standardized scanning protocol, using 128 × 3-mm section collimation, 75-millisecond rotation time, and 120-kV tube voltage. Coronary calcium lesions were defined as having an attenuation threshold greater than or equal to 130 Hounsfield units (HU) and an area greater than or equal to 1 mm^2^. Images were analyzed with the system’s integrated software (Syngo.Via; Siemens Healthcare). The total calcium burden in the coronary arteries was quantified by the scoring algorithm proposed by Agatston et al.^13^ The products of the area of each calcified plaque and peak attenuation, defined as 1 (130-199 HU), 2 (200-299 HU), 3 (300-399 HU), and 4 (≥400 HU), were summed for the left main coronary artery, left anterior descending coronary artery, left circumflex coronary artery, and right coronary artery to determine the total CAC score.

### Exposure Prediction

The method for estimating long-term outdoor air pollution concentrations for each study participant has been described in detail elsewhere.^14^ In brief, we developed a hierarchical land-use regression modeling approach for fine particulate matter less than 2.5 μm in aerodynamic diameter (PM_2.5_) and nitrogen dioxide (NO_2_) on the basis of annual mean daily monitoring data (2014-2015) from 1419 regulatory monitors nationwide. The models incorporated a wide diversity of geographic features (eg, traffic network, point of interest, population density, and land use), satellite-derived PM_2.5_ or NO_2_ observations at ground level calibrated by chemical transport model estimates, and meteorological data, with regression residuals smoothed by universal kriging. The performance of these models ranged from good to excellent, as assessed by the cross-validation (out-of-sample validation; *R*^2^ = 0.73-0.89). The model resolution was finer than 1 × 1 km^2^ for the entire country of China. Residence-specific annual mean PM_2.5_ and NO_2_ concentrations were estimated for 2015 and were used as the primary exposure measures. For O_3_, we estimated annual mean concentrations by interpolation of O_3_ observations from the proximal O_3_ monitoring sites using ordinary kriging. As an alternative assessment of traffic-related associations, we assessed residential proximity to traffic, estimated by the distance to any of the nearest roadways on a natural logarithm scale,^15^ as a secondary exposure variable. This variable is a proxy of exposure to traffic mixtures, including tailpipe and nontailpipe emissions and roadway noise.

In the sensitivity analysis, we tested different exposure time windows to our outcome. For PM_2.5_, we estimated cumulative exposures back for 3-, 4-, 5-, 6-, and 10-year periods before 2015. This analysis took advantage of our single-year fine-scale land-use regression predictions together with the 10-year temporal trend (2004-2014) generated from national satellite-derived data^16^ (eMethods in the Supplement). For NO_2_ and O_3_, a monitoring period averaged between 2014 and 2015 was examined.

### Statistical Analysis

We fit multiple linear regression models in analyzing associations of long-term air pollution exposure (PM_2.5_, NO_2_, and O_3_) and proximity to traffic with the CAC score in separate, single-pollutant models. The CAC score was modeled on a continuous scale using a logarithm (CAC score + 1) transformation to account for skewness of the distribution.

We developed statistical models in stages, by incrementally increasing control for a large set of covariates according to a priori knowledge from previous studies.^8,17^ We also used a change-in-estimate criterion by including variables if they changed the effect estimates for the unadjusted association between air pollutants and CAC by more than 10%. The primary model included the following individual-level variables: age, sex, body mass index, smoking (status, duration, and intensity), alcohol consumption, education, and physical activity. To address potential behavioral confounders that differed by geography, we also added area-level variables, including urbanization (≥2500 population per 1 × 1-km^2^ grid), study region (ie, north, southeast, and southwest), Beijing residence (yes or no), and categories of residence distances to Fuwai Hospital (<30, 30-150, 151-300, 301-500, and >500 km) in the primary model. Potential effect modification was evaluated for specific participant characteristics (age and sex), disease risk factors (body mass index, smoking, diabetes, and statin use), and geography (region and urbanization), which have been suggested to act as effect modifiers in prior studies.^7,8,9,10,18^ Because cardiovascular disease mortality risk increases in women after menopause,^3^ in an additional analysis, we restricted our analysis to postmenopausal women. To assess exposure-response associations, we refit the models using natural regression splines. To explore nonlinearity associations, we categorized exposure estimation by quartiles.

In sensitivity analyses, we used the same model but restricted analysis to participants with predicted annual exposure concentrations lower than the national standards in China (<35 and <40 μg/m^3^ for PM_2.5_ and NO_2_, respectively) or median exposure for O_3_ (<100 μg/m^3^). Second, we added to the model high-sensitivity C-reactive protein, total cholesterol, high-density lipoprotein cholesterol, low-density lipoprotein cholesterol, and triglyceride levels in the subset of 6538 participants from whom blood samples were collected. Third, the use of lipid-lowering and antihypertensive medications was added to the models. Fourth, individual-level data that included detailed information on the built environment, socioeconomic status (eg, income and employment status), and residential moving history were available for a subset of 1850 participants who completed an additional environmental survey. The sensitivity of our primary model estimates to the addition of each or a group of those covariates was also assessed. Fifth, to account for possible spatial clustering of the outcome, we used a mixed-effects model with a random effect for the 6-digit postal code (n = 917). Sixth, to assess the robustness of effect estimates in the primary model, we estimated the associations in a subset after excluding the most influential participants (Cook distance > 4/total sample size). Seventh, as noted already, we examined the effects of replacing our primary exposure variables with exposures of longer durations (2-year means for NO_2_ and O_3_, and 2-, 3-, 4-, 5-, 6-, and 10-year means for PM_2.5_). Eighth, multipollutant models were used to assess the independent effects of all the air pollutants and distance variable.

To compare with similar studies,^6,19,20^ we also assessed the association using 2 binary categorizations of CAC—detectable (CAC score >0 Agatston units) or severe (CAC score >400 Agatston units)—in logistic regression models. The presence and severity of CAC represent underlying coronary atherosclerosis in incipient and advanced stages.^21^ R statistical software version 3.4.2 (R Foundation) was used for developing the pollution exposure models, and SAS statistical software version 9.4 (SAS Institute) was used for the health model analyses. The statistical significance threshold was .05, and all tests were 2-sided.

## Results

### Study Participants and Environmental Exposures

Of the 8867 participants with a CAC score, 8168 had estimated outdoor residential pollutant concentrations for the year of the baseline examination. The participants’ mean (SD) age was 56.9 (10.4) years; 4378 (53.6%) were men (Table). More than 30% were smokers, 14.5% had at least a college education, 49.0% had diagnosed hypertension, and 5.0% had moved residence in the last 5 years.

**Table.** Descriptive Statistics of the Study Population at Baseline Classified by High and Low Estimated PM_2.5_ and NO_2_ Concentrations in 2015 in China

Characteristic	Entire Cohort (N = 8168)^a^	PM_2.5_, μg/m^3^		NO_2_, μg/m^3^		*P* Value^b^
		≤80 (n = 4250)^a^	>80 (n = 3918)^a^	≤42 (n = 4083)^a^	>42 (n = 4085)^a^	
Coronary artery calcium present	3676 (45.0)	1828 (43.0)	1848 (47.0)	1748 (42.8)	1928 (47.2)	
Coronary artery calcium score, Agatston units						
Mean (SD)	91.4 (322.2)	84.9 (304.5)	98.6 (340.6)	87.9 (325.4)	94.9 (319.0)	
1-100	1986 (24.3)	985 (23.2)	1001 (25.5)	932 (22.8)	1054 (25.8)	
101-300	896 (11.0)	458 (10.8)	438 (11.2)	449 (11.0)	447 (10.9)	
>300	794 (9.7)	385 (9.0)	409 (10.3)	367 (9.0)	427 (10.5)	
Demographic characteristics						
Age, mean (SD), y	56.9 (10.4)	56.7 (9.8)	57.1 (11)	56.6 (9.6)	57.1 (11.1)	<.001
Male	4378 (53.6)	2163 (50.9)	2215 (56.5)	2086 (51.1)	2292 (56.1)	<.001
Education						
<College	6984 (85.5)	3919 (92.2)	3065 (78.2)	3838 (94)	3146 (76.9)	<.001
College	1045 (12.8)	299 (7.0)	746 (19.1)	233 (5.7)	812 (20.0)	
>College	139 (1.7)	32 (0.8)	107 (2.7)	12 (0.3)	127 (3.1)	
Risk factors						
BMI, mean (SD)	25.4 (3.2)	25.3 (3.2)	25.6 (3.3)	25.3 (3.3)	25.5 (3.2)	.01
Smoking status						<.001
Never and former	5652 (69.2)	2994 (70.5)	2658 (67.9)	2864 (70.2)	2788 (68.3)	
Current	2516 (30.8)	1256 (29.5)	1260 (32.1)	1219 (29.8)	1297 (31.7)	
Cigarettes per/d						
≤10	1242 (15.2)	606 (14.2)	636 (16.2)	558 (13.6)	684 (16.7)	<.001
11-20	4990 (61.1)	2614 (61.6)	2376 (60.7)	2520 (61.8)	2470 (60.6)	
>20	1936 (23.7)	1030 (24.2)	906 (23.1)	1005 (24.6)	931 (22.7)	
Smoking duration, mean (SD), y	28.5 (10.3)	28.9 (10)	28.1 (10.6)	29 (10.1)	27.9 (10.5)	<.001
Alcohol consumption	1764 (21.6)	852 (20.1)	912 (23.3)	820 (20.1)	944 (23.1)	<.001
Physical activity, times per/wk						
Never	1413 (17.3)	737 (17.3)	676 (17.2)	723 (17.7)	690 (16.9)	<.001
≤3	3994 (48.9)	2074 (48.8)	1920 (49.0)	2007 (49.1)	1987 (48.6)	
>3	2761 (33.8)	1439 (33.9)	1322 (33.8)	1353 (33.2)	1408 (34.5)	
Hypertension	4002 (49.0)	2052 (48.3)	1950 (49.8)	2009 (49.2)	1993 (48.8)	<.001
Diabetes	1299 (15.9)	614 (14.5)	685 (17.5)	572 (14.0)	727 (17.8)	<.001
Coronary artery disease^c^	1715 (21.0)	927 (21.8)	788 (20.1)	898 (22.0)	817 (20.0)	<.001
Medication						
Antihypertensive	3725 (45.6)	1917 (45.1)	1808 (46.1)	1892 (46.3)	1833 (44.8)	<.001
Statin use	2156 (26.4)	1041 (24.5)	1115 (28.4)	1004 (24.6)	1152 (28.2)	<.001
Menopausal women	1732 (45.7)	823 (41.3)	909 (50.6)	791 (40.0)	941 (52.0)	
Geography						
Urban	5628 (68.9)	2309 (54.4)	3319 (84.8)	1870 (45.8)	3758 (92.0)	.02
Region						
North	6690 (81.9)	2979 (70.1)	3711 (94.7)	2774 (67.9)	3916 (95.8)	.05
Southeast	939 (11.5)	732 (17.3)	207 (5.3)	815 (20.0)	124 (3.1)	
Southwest	539 (6.6)	539 (12.6)	0	494 (12.1)	45 (1.1)	
Participants with blood samples obtained, No.	6528	3645	2883	3516	3012	
Blood markers, mean (SD)						
Cholesterol, mg/dL						
Total	181.5 (50.2)	181.5 (46.3)	181.5 (50.2)	181.5 (46.3)	181.5 (50.2)	<.01
High-density lipoprotein	36.4 (33.6)	33.6 (14.0)	36.4 (50.4)	33.6 (14.0)	36.4 (47.6)	.04
Low-density lipoprotein	84.0 (81.2)	81.2 (28.0)	84.0 (120.4)	81.2 (28.0)	84.0 (117.6)	.08
Triglycerides, mg/dL	150.4 (115.0)	159.3 (123.9)	150.4 (106.2)	159.3 (123.9)	150.4 (106.2)	.06
High-sensitivity C-reactive protein, mg/L	1.9 (4.4)	2 (5.5)	1.8 (2.5)	2 (5.6)	1.7 (2.5)	.05
Moved residence in the last 5 y^d^	93 (5.0)	47 (5.1)	46 (4.9)	42 (4.8)	51 (5.1)	

Abbreviations: BMI, body mass index (calculated as weight in kilograms divided by height in meters squared); NO_2_, nitrogen dioxide; PM_2.5_, particulate matter with aerodynamic diameter less than 2.5 μm.

SI conversion factors: To convert total, high-density lipoprotein, and low-density lipoprotein cholesterol to millimoles per liter, multiply by 0.0259; triglycerides to millimoles per liter, multiply by 0.0113; and high-sensitivity C-reactive protein to nanomoles per liter, multiply by 9.524.

^a^ Except where noted otherwise, data are number (percentage) of participants.

^b^ *P* value from univariate regression between coronary artery calcium and individual variables.

^c^ Defined as 50% or more stenosis in 1 or more major epicardial vessels.

^d^ Data are based on subpopulation of 1850 participants who had completed detailed information.

**Figure 2.**
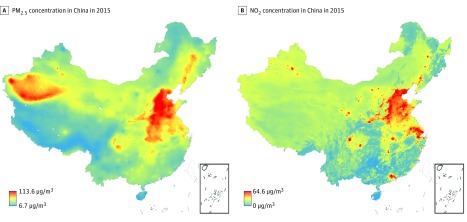
Spatial Distribution of Estimated Annual Pollution Concentrations in 2015 in China A, Concentration of particulate matter with aerodynamic diameter less than 2.5 μm (PM_2.5_) per 1 × 1 km^2^_. _B, Concentration of nitrogen dioxide (NO_2_) per 1 × 1 km^2^.

Annual mean PM_2.5_, NO_2_, and O_3_ measurements were 70.1 (20.0), 41.4 (14.7), and 93.9 (10.5) μg/m^3^, respectively. Annual mean air pollutant concentrations varied substantially among the study participants (eTable 1 in the Supplement), with all pollutants being highest for participants in the Hebei province and lowest for those in the south (Figure 2; eFigure 1 in the Supplement). The participants were exposed to higher concentrations of PM_2.5_ and NO_2_ in urban areas than in rural areas. Predictions for PM_2.5_ were positively correlated with NO_2_ and O_3_ (Spearman correlation coefficients, 0.72 and 0.53, respectively) and negatively correlated with proximity to a roadway (Spearman correlation coefficient, −0.10). Of the participants, 7728 (95.0%) had estimated exposures greater than 35 μg/m^3^ for PM_2.5_ and 4551 (56.0%) had estimated exposures greater than 40 μg/m^3^ for NO_2_.

### Air Pollution, Proximity to Traffic, and CAC

**Figure 3.**
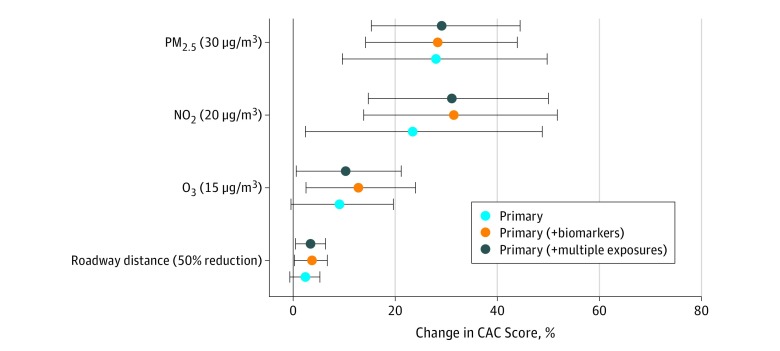
Coronary Artery Calcium (CAC) Score Associated With an Increase in Long-term Air Pollutant Exposure Percentage increase in CAC scores with 95% CIs (error bars). The interquartile range increases in pollutants are 30 μg/m^3^ for particulate matter with aerodynamic diameter less than 2.5 μm (PM_2.5_), 20 μg/m^3^ for nitrogen dioxide (NO_2_), and 15 μg/m^3^ for ozone (O_3_); there was a 50% decrease for distance from a roadway. The primary model included age, sex, body mass index, smoking status, smoking years, cigarettes per day, alcohol consumption, education, exercise, urbanization, regions, distance to hospital, and Beijing residence (yes or no). The primary model plus biomarkers also included levels of total cholesterol, high-density lipoprotein cholesterol, low-density lipoprotein cholesterol, triglycerides, and high-sensitivity C-reactive protein. The primary model plus multiple exposures also included PM_2.5_, NO_2_, O_3_, and distance to road variables.

The mean (SD) CAC score was 91.4 (322.2) Agatston units. Long-term exposure to PM_2.5_, NO_2_, and O_3_, as well as distance to the nearest roadway, were all associated with higher CAC score in the crude, moderately adjusted, and fully adjusted single-pollutant models (fully adjusted single-pollutant model, PM_2.5_ per 30 μg/m^3^: 29.6%; 95% CI, 15.7%-45.2%; NO_2_ per 20 μg/m^3^: 33.2%; 95% CI, 16.4%-52.4%; O_3_ per 15 μg/m^3^: 10.8%; 95% CI, 0.9%-21.8%; distance to roadway per 50% decrease: 3.2%; 95% CI, 0.3%-6.2%) (Figure 3; eTable 2 in the Supplement). Independent association with CAC score was 9.0% (95% CI, −1.4% to 20.4%) for O_3_ per 15 μg/m^3^ and 2.4% (95% CI, −0.6% to 5.4%) for distance near roadway per 50% decrease. Associations were not substantially changed by additional adjustment for medications and blood biomarkers, accounting for neighborhood clustering, use of alternative exposure durations, and exclusion of data with extreme observations (eTable 2 and eFigure 2 in the Supplement). The addition of individual home environment variables (eg, environmental tobacco smoking, use of air conditioner, air purifier, cooking stoves, or a ventilation system) in a subgroup analysis of 1850 participants, the addition of socioeconomic status variables (eg, income and employment status), or the exclusion from the subgroup analysis of 88 participants who had moved similarly did not meaningfully change the estimates (eTable 3 in the Supplement).

When we restricted the analyses to the subset of participants with predicted exposure concentrations below the national standard (or median of exposure distribution), the association between NO_2_ and CAC was statistically significant (NO_2_ per 10 μg/m^3^: 24.0%; 95% CI, 6.4% to 44.5%). The association with O_3_ was not significant (O_3_ per 10 μg/m^3^: 7.4%; 95% CI, −0.5% to 16.1%). The association between PM_2.5_ and CAC in postmenopausal women was significant (PM_2.5_ per 10 μg/m^3^: 34.5%; 95% CI, 5.8% to 70.9%).

In the multipollutant models with all exposure variables included, the effect estimates for both PM_2.5_ and NO_2_ were largely unchanged, with a 27.2% (95% CI, 10.8%-46.1%) greater CAC score for a 30-μg/m^3^ increase in PM_2.5_ and a 24.5% (95% CI, 3.6%-49.7%) greater CAC score per 20-μg/m^3^ increase in NO_2_. The concentration-response curve suggested little evidence of a nonlinear association with either PM_2.5_ or NO_2_, (eFigure 3 in the Supplement). Estimating associations by quartiles of the pollutants confirmed the reasonably linear nature of the concentration-response curves (eFigure 4 in the Supplement).

**Figure 4.**
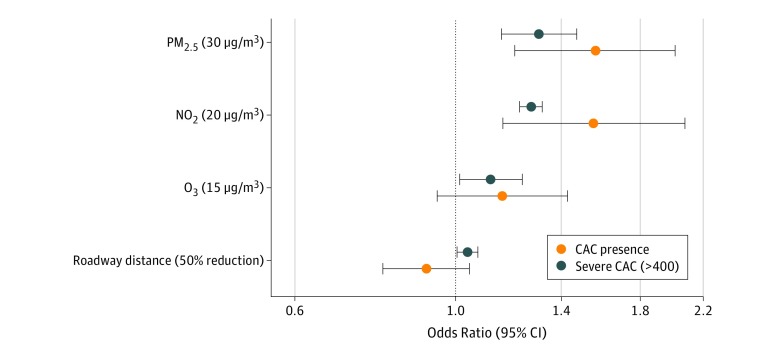
Odds Ratio of Detectable and High-Level Coronary Artery Calcium (CAC) Associated With Long-term Exposure Variables Odds ratios and 95% CIs (error bars) for the presence of CAC and severe CAC (>400 Agatston units) adjusted for age, sex, body mass index, smoking status, smoking years, cigarettes per day, alcohol consumption, education, exercise, urbanization, region, distance to hospital, and Beijing residence (yes or no). NO_2_ indicates nitrogen dioxide; O_3_, ozone; and PM_2.5_, particulate matter with aerodynamic diameter less than 2.5 μm.

In the logistic regression analyses, there were higher odds of the presence of CAC with increasing exposures to all air pollutants and distance to roadway variables (PM_2.5_ per 30 μg/m^3^: odds ratio [OR], 1.28; 95% CI, 1.13-1.45; NO_2_ per 20 μg/m^3^: OR, 1.27; 95% CI, 1.23-1.32; O_3_ per 15 μg/m^3^: OR, 1.12; 95% CI, 1.01-1.24; distance to roadway per 50% decrease: OR, 1.04; 95% CI, 1.00-1.07) (Figure 4). Also, the associations with severe CAC scores were greater in participants with increased exposure to PM_2.5_ (OR, 1.59; 95% CI, 1.20-2.12) or NO_2_ (OR, 1.60; 95% CI, 1.18-2.17).

### Modification of Associations

We observed stronger associations between air pollutants (PM_2.5_ and NO_2_) and CAC score in men (PM_2.5_: 42.2%; 95% CI, 24.3% to 62.7%; NO_2_: 45.7%; 95% CI, 25.3% to 69.5%) and older (age >60 years) participants (PM_2.5_: 50.1%; 95% CI, 28.8% to 75.0%; NO_2_: 55.5%; 95% CI, 31.8% to 83.6%) (eTable 4 in the Supplement). Participants with diabetes had larger NO_2_ association estimates than did participants without diabetes (PM_2.5_: 62.2%; 95% CI, 30.9% to 101.0%; NO_2_: 31.2%; 95% CI, 13.9% to 51.0%), and participants in the north had larger association estimates than did participants in the southeast regions (NO_2_ per 20 μg/m^3^, north region: 42.0%; 95% CI, 21.7% to 65.6%; southeast region: −9.2%; 95% CI, −32.1% to 21.4%). There were no clear differences in the pollutant association estimates between local Beijing and non-Beijing residents (local Beijing residents, per 1-SD increase in PM_2.5_: 10.3%; 95% CI, 1.8% to 19.4%; NO_2_: 9.2%; 95% CI, 0.9% to 18.3%; and O_3_: −3.2%; 95% CI, −11.5% to 6.0%; and non-Beijing residents, per 1-SD increase in PM_2.5_: 16.1%; 95% CI, 9.4% to 23.1%; NO_2_: 14%; 95% CI, 7.1% to 21.4%; O_3_: 9.9%; 95% CI, 3.5% to 16.7%) (eTable 5 in the Supplement).

## Discussion

In this large Chinese study, long-term exposures to PM_2.5_, NO_2_, and O_3_ and proximity to traffic were associated with subclinical coronary atherosclerosis as assessed by the CAC score in residents with potential risk of CHD. The findings for PM_2.5_ and NO_2_ were robust to the use of various exposure durations and to controlling for major known CHD risk factors and copollutants. The associations were stronger among men, elderly participants, and those with diabetes. Given that CAC is strongly associated with total CHD events,^4^ coupled with the recently reported finding that higher long-term PM_2.5_ exposure was associated with a higher risk of CHD mortality in China,^10^ this study may provide evidence suggesting that coronary atherosclerosis is a pathological pathway through which air pollution exposure potentially increases mortality associated with CHD.

Major strengths of our study include the large population sample size, the use of advanced methods for estimating individual-level long-term outdoor exposure concentrations, and the high quality of individual information on both the outcome measures and potential confounding factors. Compared with numerous epidemiological studies in China that estimated air pollution exposures from either proximal monitors^22^ or regional modeling estimates (≥10 × 10-km spatial resolution),^10^ our exposure models produced individually resolved exposure estimates that allowed us to use accurate fine-scale contrast in investigating the association between air pollution exposure and atherosclerosis.

The associations identified in our study were likely more apparent than those in prior studies owing to the very broad range of ambient air pollution concentrations we observed. In addition, this is a population at risk for coronary atherosclerosis. Prior studies^6,9,19,20^ showing an association of air pollution or traffic exposure with the degree of CAC were very limited, with mostly null findings. A higher degree of CAC was associated with exposure to nearby roadways in the Ruhr area of Germany,^9^ and an association between PM_2.5_ and thoracic aortic calcifications was recently reported elsewhere.^17^ In the Multi-ethnic Study of Atherosclerosis,^8^ higher exposures to PM_2.5_ and NO_2_ were associated with faster progression of CAC, but cross-sectional associations, such as those seen in this study, have not been observed.^6^ Compared with previous studies of calcification in less-polluted regions, the observed magnitude of our estimates seemed smaller for PM_2.5_ (eFigure 5 in the Supplement). This is in line with a recent Global Burden of Diseases analysis^1^ indicating a steeper change in relative risk for countries at much lower levels of PM_2.5_ compared with those at higher values.

In 2015, more than 95% of the Chinese population was exposed to concentrations of PM_2.5_ and NO_2_ greater than the minimum level of our study.^14^ Because more than 40% of all deaths in China are attributable to cardiovascular disease,^23^ the potential contribution of air pollutants to the burden of cardiovascular disease in China is very large. Improving air quality to, for example, the Chinese national standards of 35 μg/m^3^ for PM_2.5_ and 40 μg/m^3^ for NO_2_ may lead to a longer life span.^24^ The association with NO_2_ exposure on CAC persisted even when the analysis was restricted to concentrations less than 40 μg/m^3^, suggesting that associations may occur even below this level.

In our study, we also found that long-term exposure to PM_2.5_ and NO_2_ was associated with increased likelihood of both the presence of detectable CAC at any amount, and the most severe levels of CAC; the risk of severe CAC was most pronounced. This is consistent with 2 US studies^25,26^ that observed higher risks of mild and severe CHD associated with exposure to PM_2.5_. Together, these findings and the results of our study indicate that individuals at potential risk for CHD are a population that is especially susceptible to air pollution exposures.

Mechanisms by which PM_2.5_ exposure affects atherogenesis are not well elucidated. It has been proposed that the generation of oxidative reaction products is initiated by the reaction of pollutants with lipids or cellular membranes in the airways and lung alveoli, with circulating products triggering atherogenic mechanisms such as lipid peroxidation and high-density lipoprotein dysfunction.^3^ Persistent activation of this pathway is associated with the development of atherosclerosis.^27^ We cannot conclude from our study that NO_2_ per se is the specific pollutant responsible for these estimated effects. Nitrogen dioxide may simply be an indicator of more harmful traffic mixtures, including ultrafine particles and diesel exhaust black carbon. Diesel exhaust and ultrafine particles have been shown to elicit harmful biological responses in experimental models.^28^ Indeed, we observed that exposure to NO_2_ was associated with CAC and that adjusting for roadway proximity attenuated its association with NO_2_. Living close to a roadway is associated with a substantial increase an individual’s exposure to traffic-related air pollutants, including tailpipe (eg, NO_2_, ultrafine particles, and black carbon) and nontailpipe (eg, particles from tire wear and friction materials) emissions and noise.^29^ This finding suggests that the synergistic effect of traffic exposure mixtures might, at least in part, operate through this mechanism.

Prior evidence^8^ has supported the role of PM_2.5_ and traffic-related air pollutants such as NO_2_ in the development of atherosclerosis, but less research has focused on the cardiovascular effects of O_3_; the long-term effect of exposure to O_3_ on subclinical CHD is still largely unknown. Ambient O_3_ is a powerful oxidizing agent and common air pollutant worldwide. We observed a significant association between O_3_ and degree of CAC, although this association could not be disentangled from the effects of PM_2.5_ and NO_2_ because of the spatial correlations between these exposure variables.

There was evidence that elderly participants and those with diabetes were more susceptible to these exposure effects than other participants. Patients with diabetes have a greater overall coronary plaque burden and a higher rate of multivessel disease. It is possible that air pollution could worsen the underlying diabetes disease course by exacerbating insulin resistance or by instigating adverse biological responses (eg, endothelial dysfunction) that promote future diabetic atherosclerosis.^3^ Among women, we observed a stronger association of exposure to PM_2.5_ with risk of atherosclerosis in postmenopausal women, a group for whom an association between PM_2.5_ and cardiovascular risk has previously been demonstrated in the United States.^30^

### Limitations

This study has several limitations. First, because this was an outpatient population, it may not fully reflect the general population. However, this population had relatively low pretest probability of CHD (27.3%); 29.0% had no symptoms, and 64.0% had nonanginal chest pain. Moreover, this population resembled population-based samples^8,10,17^ in terms of risk factors and CAC levels. Therefore, this cohort can represent a low-risk natural population. Second, we cannot rule out biases due to unmeasured confounders, such as occupation history. In addition, the main analysis of this study did not account for individuals who moved in recent years, although we did sensitivity analysis in a subset of the samples that showed consistent association. Third, this is a cross-sectional study involving the baseline examination of an ongoing prospective cohort study. Future longitudinal analysis of this prospective cohort may provide additional information.

## Conclusions

This study found that long-term exposure to outdoor air pollution and traffic pollutants in China, specifically PM_2.5_ and NO_2_, was associated with the development of CAC. This finding should contribute to an understanding of air pollutant effects worldwide, providing both much needed locally generated data and supportive evidence to inform the air pollution standard-setting process on a global scale.

## Notes

Supplement. **eMethods. **Supplemental Methods **eTable 1. **Summary Statistics and Correlations of the Exposure Variables in the Study Participants **eTable 2. **Main and Sensitivity Analyses for Percent Change and 95% CI in CAC Associated With a Difference in Long-term Exposure Variables Among the CREATION Participants **eTable 3. **Sensitivity Analyses With Detailed Individual-Level Risk Factors for Associations Between Long-term Air Pollution Exposure Variables and CAC-score Among a Subset of Participants (n = 1850) **eTable 4. **Effect Modifications in Associations of CAC With Long-term Exposure to PM_2.5_ and NO_2_ for Selected Demographics and Risk Factors **eTable 5. **Comparisons of Health Effect Estimates in and Outside Beijing **eFigure 1. **Spatial Distribution of Estimated Annual Mean Exposure to PM_2.5_, NO_2_, and O_3_ in the Year of 2015 at the Participants’ Home and Regional Classifications **eFigure 2. **Percent Change and 95% CI in CAC Associated With PM_2.5_ (30 µg/m^3^) Averaged Over Multiple Years Prior to Baseline Using Land Use Regression Model With Adjustment for Satellite-derived PM_2.5_ Observations **eFigure 3. **Concentration-Response Relationships Between Long-term Exposure to PM_2.5_ and NO_2_ and the Estimated Percent Change in CAC Score, Modeled by a Natural Spline With 3 Degrees of Freedom **eFigure 4. **Associations Between CAC and Long-term Exposure Variables Categorized by Quartiles **eFigure 5. **Comparisons of Heath Effect Estimates for Associations of PM_2.5_ and Proximity to Traffic Exposures With Degrees of CAC, Thoracic Aortic Calcification (TAC) or Abdominal Aortic Calcification (AAC) Between This Study and the Previous Studies **eReferences**
