# Supplementary material for: Association of Estimated Long-term Exposure to Air Pollution and Traffic Proximity With a Marker for Coronary Atherosclerosis in a Nationwide Study in China
Source: JAMA Netw Open. 2019 Jun 28;2(6):e196553. doi: 10.1001/jamanetworkopen.2019.6553 (PMC6604100; doi:10.1001/jamanetworkopen.2019.6553)
Supplement: Supplement. — eMethods. Supplemental Methods eTable 1. Summary Statistics and Correlations of the Exposure Variables in the Study Participants eTable 2. Main and Sensitivity Analyses for Percent Change and 95% CI in CAC Associated With a Difference in Long-term Exposure Variables Among the CREATION Participants eTable 3. Sensitivity Analyses With Detailed Individual-Level Risk Factors for Associations Between Long-term Air Pollution Exposure Variables and CAC-score Among a Subset of Participants (n = 1850) eTable 4. Effect Modifications in Associations of CAC With Long-term Exposure to PM2.5 and NO2 for Selected Demographics and Risk Factors eTable 5. Comparisons of Health Effect Estimates in and Outside Beijing eFigure 1. Spatial Distribution of Estimated Annual Mean Exposure to PM2.5, NO2, and O3 in the Year of 2015 at the Participants’ Home and Regional Classifications eFigure 2. Percent Change and 95% CI in CAC Associated With PM2.5 (30 µg/m3) Averaged Over Multiple Years Prior to Baseline Using Land Use Regression Model With Adjustment for Satellite-derived PM2.5 Observations eFigure 3. Concentration-Response Relationships Between Long-term Exposure to PM2.5 and NO2 and the Estimated Percent Change in CAC Score, Modeled by a Natural Spline With 3 Degrees of Freedom eFigure 4. Associations Between CAC and Long-term Exposure Variables Categorized by Quartiles eFigure 5. Comparisons of Heath Effect Estimates for Associations of PM2.5 and Proximity to Traffic Exposures With Degrees of CAC, Thoracic Aortic Calcification (TAC) or Abdominal Aortic Calcification (AAC) Between This Study and the Previous Studies eReferences [file jamanetwopen-2-e196553-s001.pdf]

Supplementary Online Content

Wang M, Hou Z-H, Xu H, et al. Association of estimated long-term exposure to air pollution and traffic proximity with a marker for coronary atherosclerosis in a nationwide study in China. *JAMA Netw Open*. 2019;2(6):e196553.
doi:10.1001/jamanetworkopen.2019.6553

eMethods. Supplemental Methods

eTable 1. Summary Statistics and Correlations of the Exposure Variables in the Study Participants

eTable 2. Main and Sensitivity Analyses for Percent Change and 95% CI in CAC Associated With a Difference in Long-term Exposure Variables Among the CREATION Participants

eTable 3. Sensitivity Analyses With Detailed Individual-Level Risk Factors for Associations Between Long-term Air Pollution Exposure Variables and CAC-score Among a Subset of Participants (n = 1850)

eTable 4. Effect Modifications in Associations of CAC With Long-term Exposure to $PM_{2.5}$ and NO_2 for Selected Demographics and Risk Factors

eTable 5. Comparisons of Health Effect Estimates in and Outside Beijing

eFigure 1. Spatial Distribution of Estimated Annual Mean Exposure to $PM_{2.5}$, NO_2, and O_3 in the Year of 2015 at the Participants' Home and Regional Classifications

eFigure 2. Percent Change and 95% CI in CAC Associated With $PM_{2.5}$ (30 μg/m^3) Averaged Over Multiple Years Prior to Baseline Using Land Use Regression Model With Adjustment for Satellite-derived $PM_{2.5}$ Observations

eFigure 3. Concentration-Response Relationships Between Long-term Exposure to $PM_{2.5}$ and NO_2 and the Estimated Percent Change in CAC Score, Modeled by a Natural Spline With 3 Degrees of Freedom

eFigure 4. Associations Between CAC and Long-term Exposure Variables Categorized by Quartiles

eFigure 5. Comparisons of Heath Effect Estimates for Associations of $PM_{2.5}$ and Proximity to Traffic Exposures With Degrees of CAC, Thoracic Aortic Calcification (TAC) or Abdominal Aortic Calcification (AAC) Between This Study and the Previous Studies

eReferences

This supplementary material has been provided by the authors to give readers additional information about their work.

© 2019 Wang M et al. *JAMA Network Open.*

eMethods. Supplemental Methods

1. Description of the study cohort

1.1. Study population

The Coronary Atherosclerosis Disease Early Identification and Risk Stratification by Noninvasive Imaging (CREATION, clinical trial registration number: NCT03518437) study is a large and newly developed cohort study with participants selected from outpatients nationwide primarily from north, southwest and southeast of China. This cohort was organized by Beijing Fuwai hospital affiliated to Chinese Academy of Medical Sciences and National Center for Cardiovascular Diseases of China, with major focus on identifying risk factors that lead to coronary artery atherosclerotic plaque progression and events of coronary artery diseases (CAD). Fuwai Hospital is the largest hospital in China, specializing cardiovascular diseases treatment, prevention and research. Nowadays, more than 6 million outpatients, from 32 provinces in the nation and 6 countries worldwide have received their cares.

The CREATION cohort recruited outpatients of adults (aged>25) that visit Fuwai hospital who are suspected of having CAD without history of coronary revascularization other heart disease and who underwent cardiac computed tomography between 2015 and 2017. Similar study design could be found elsewhere[1]. The participants had relatively low pretest probability of CHD (27.3±15.1%), with asymptomatic patients (n=2587), non-anginal chest pain (n=5721), atypical angina (n=449), typical angina (n=110), therefore, this cohort can represent the low risk natural population. Typical angina was defined as having all of the following criteria: (1) substernal chest pain or discomfort that was (2) provoked by exertion or emotional stress and (3) relieved by rest and/or nitroglycerine. Symptoms were classified as atypical angina when only two of these criteria were present, and as nonanginal chest pain if one or none of these characteristics were reported.

This study built air pollution and traffic exposures upon the CREATION cohort participants who had accurate geocoded addresses for exposure assessment at baseline. All the participants will be followed-up for CAD disease events collection and some of them will be re-examined for CT scans after 3 and 5 years of baseline survey. In addition to clinical information collected during the hospital visit and subsequent visits for follow-up treatment or physical examinations, an extensive set of cardiovascular biomarkers of was collected in this cohort. Furthermore, a subset of the participants completed detailed survey on environmental risk factors, including building environment, socio-economic status and residential mobility. The large cohort size and rich subclinical and biological database make it feasible to associate long-term air pollution and traffic exposures with outcomes that may define pathways by which air pollution contributes to the atherosclerotic cardiovascular disease. Detailed participants selection and spatial distributions of their residences exposed to air pollution were shown as below.

1.2. Clinical data

A structured interview was conducted before CT exam to collect information on demographic characteristics, the presence and duration of cardiovascular risk factors and biochemical results. Hypertension was defined as a previously established diagnosis, systolic blood pressure ≥140 mmHg, diastolic blood pressure ≥90 mmHg, or antihypertensive medication use. Hypercholesterolemia was defined according to the National Cholesterol Education Panel (NCEP) guidelines or by the current use of lipid-lowering medication. Diabetes mellitus was defined as a previously established diagnosis, insulin or oral hypoglycemic therapy. Current smoking was defined as any cigarette smoking within 1 year prior to the CT exam. Alcohol consumption was defined as drinking more than twice a week for more than a year. Meat consumption was defined as eating meat more than three times a week, more than 300 grams for each time. Physical activity was defined as frequency of aerobic exercise for more than an hour. Coronary heart disease was defined as at least 1 coronary segment with a lesion of ≥50% luminal stenosis in diameter. Any disagreements between two readers were solved by consensus.The duration of the risk factors was defined as the time between made a definite

© 2019 Wang M et al. *JAMA Network Open.*

diagnosis and underwent CT. The results of the biochemical test were performed within 1 month of the CT exam.

2. Estimating historical $PM_{2.5}$

To extrapolate $PM_{2.5}$ concentrations back in time, we leveraged a large dataset with annual average satellite-derived $PM_{2.5}$ estimates (10x10 km^2) from 2004 to 2014[2]. We converted our fine-scale point-based PM2.5 predictions in 2014 to the historical years using the ratio of the satellite-derived $PM_{2.5}$ predictions between 2014 and the target year (see equation 1). This calculation is based on an assumption that, for each point-based prediction from our fine-scale model, the spatial contrast of $PM_{2.5}$ is constant between the two years within a 10x10km grid cell, but varies between grid cells. We then calculated the long-term $PM_{2.5}$ concentrations averaged back for 3-, 4-, 5-, 6-, and 10-year respectively, ahead of baseline since 2015, to assess the effect of different lengths of exposure on difference of CAC.

$$PM2.5_{year(i)}^{LUR(point)} = PM2.5_{2014}^{LUR(point)} \times \frac{PM2.5_{year\ (i)}^{SAT(10km)}}{PM2.5_{2014}^{SAT(10km)}} \quad \text{(Equation 1)}$$

Where year (i) donates the target year to be extrapolated to between 2004 and 2014, SAT donates the 10x10 km^2 annual averaged satellite-derived $PM_{2.5}$ estimates, and LUR donates our point-based hierarchical land use regression modeling predictions.

eTable 1 Summary Statistics and Correlations of the Exposure Variables in the Study Participants

Exposure	Min	Q1	Median	Q3	Max	Correlation coefficients			
						$PM_{2.5}$	NO_2	O_3	Distance to roadway (feet)
$PM_{2.5}$ (μg/m^3)	17.5	52.9	80.4	85.5	108.0	1	0.72	0.53	-0.1
NO_2 (μg/m^3)	8.2	29.4	42.4	54.5	62.8	0.72	1	0.56	-0.24
O_3 (μg/m^3)	49.9	88.9	98.7	101.5	122.1	0.53	0.56	1	-0.14
Distance to roadway (feet)	12.1	98.4	218.8	134.3	4955.3	-0.1	-0.24	-0.14	1

© 2019 Wang M et al. *JAMA Network Open.*

eTable 2 Main and Sensitivity Analyses for Percent Change and 95% CI in CAC Associated With a Difference in Long-term Exposure Variables[a] Among the CREATION Participants.

ID	N	Staged models[b]	$PM_{2.5}$ (30 μg/m^3)	NO_2 (20 μg/ m^3)	O_3 (15 μg/ m^3)	Distance to road (reduction by 50%)
1	8168	Model 1	16.6 (8.4, 25.5)	8.5 (1.2, 16.3)	9.4 (2.5, 16.7)	2.5 (-0.1 ,5.3)
2	8168	Model 1 (+risk factor)	17.4 (8.9, 26.5)	11 (3.1, 19.4)	8.9 (1.9, 16.4)	3.0 (0.3 ,5.8)
3	8168	Model 2 (+area-level covariates, primary model)	29.6 (15.7, 45.2)	33.2 (16.4, 52.4)	10.8 (0.9, 21.8)	3.2 (0.3 ,6.2)
4	8168	Model 3 (+medications)	27.6 (13.9, 42.8)	31.4 (14.9, 50.2)	9.5 (-0.2, 20.2)	3.0 (0.1 ,6.0)
5	6538	Model 3 (+biomarkers)	28.8 (13.8, 45.8)	31.5 (13.5, 52.3)	11.5 (0.8, 23.4)	3.1 (0.2 ,6.1)
6	8168	Model 3 (+multi-pollutants)	27.2 (10.8, 46.1)	24.5 (3.6, 49.7)	9.0 (-1.4, 20.4)	2.4 (-0.6 ,5.4)
7	8168	Model 3(spatial cluster)	30.2 (16.1, 46.0)	33.0 (16.2, 52.2)	10.5 (0.5, 21.4)	3.1 (0.2 ,6.1)
8	8015	Model 3 (excluding extreme values)	47.8 (33.0, 64.2)	57.4 (39.0, 78.3)	11.9 (2.6, 22.2)	3.2 (0.3 ,6.2)
9	8168	Model 3 (two-year average exposure)	27.4 (14.8, 41.4)	26.8 (10.8, 45.2)	9.5 (0.3, 19.5)	
10	$PM_{2.5}$: 440<br>NO_2: 3617<br>O_3: 4468	Model 3 (below national standard)	22.8 (-9.1, 66.0)	24.0 (6.4, 44.5)	7.4 (-0.5, 16.1)	
11	1732	Model 3 (postmenopausal women)	34.5 (5.8, 70.9)	26.6 (-2.8, 65.0)	4.1 (-8.5, 18.5)	

[a]Increment difference of each exposure variables for the health effect estimates are 30 μg/m^3 for $PM_{2.5}$, 20 μg/m^3 for NO_2, 15 ug/m^3 for O_3 and 50% decrease for road distance;

[b]Model 1= age, gender;
Model 2= model1+BMI, smoking status, smoking years, cigarettes per day, alcohol consumptions, education, exercise;
Model 3= model2+ urbanization, regions, distance to hospital, local Beijing (yes/no);
Model 4=model 3+ antihypertensive medicine and use of statin;
Model 5=model 3+biomarkers: total cholesterol, high and low density lipoprotein, triglycerides, and high-sensitivity C-reactive protein
Model 6=model 3+multi-pollutants: $PM_{2.5}$, NO_2, O_3, distance to roadway were modeled simultaneously.

© 2019 Wang M et al. *JAMA Network Open.*

Model 7=model 3+random slope accounting for county-level area effects;
Model 8=model 3 without extreme observations identified by cook's distance values;
Model 9=model 3 with exposure to air pollutants averaged between 2014 and 2015.
Model 10=model 3 with exposure below the national standard (i.e. <35 μg/m^3 national standard for annual average $PM_{2.5}$, <40 μg/m^3 national standard for annual average NO_2, and <100 μg/m^3 US standard for annual average O_3); increment difference of exposure for the health effect estimates are 10 μg/m^3 for both $PM_{2.5}$ and NO_2.
Model 11=model 3 restricted to postmenopausal women.

© 2019 Wang M et al. *JAMA Network Open.*

eTable 3 Sensitivity Analyses With Detailed Individual-Level Risk Factors for Associations Between Long-term Air Pollution Exposure Variables[a] and CAC-score Among a Subset of Participants (n = 1850)

Staged models	N	$PM_{2.5}$[b] (30 μg/ m^3)	NO_2 (20 μg/ m^3)	O_3 (15 μg/ m^3)	Distance to road (reduction by 50%)
Model 2[b]	1850	36.5 (14.0 ,63.4)	36.9 (8.6 ,72.6)	18.7 (1.2 ,39.1)	6.1 (-1.2 ,14.0)
Model 2+Air conditioner	1850	40.5 (17.1 ,68.6)	43.1 (13.0 ,81.2)	19.2 (1.4 ,40.1)	5.7 (-1.7 ,13.5)
Model 2+Heating	1850	39.1 (16.1 ,66.6)	41.4 (12.0 ,78.5)	18.5 (1.0 ,39.0)	6.0 (-1.4 ,13.8)
Model 2+Air purifier	1850	39.1 (16.1 ,66.6)	41.3 (11.9 ,78.4)	18.5 (1.0 ,39.0)	5.7 (-1.6 ,13.6)
Model 2+Cooking[c]	1850	38.6 (15.7 ,66)	40.9 (11.6 ,77.8)	17.5 (0.1 ,37.8)	5.5 (-1.8 ,13.4)
Model 2+Ventilation[d]	1850	37.9 (15.0 ,65.3)	40.7 (11.4 ,77.7)	18.8 (1.2 ,39.5)	5.4 (-1.9 ,13.3)
Model 2+Environmental tobacco smoking	1850	36.5 (14.1 ,63.4)	36.8 (8.5 ,72.5)	18.7 (1.2 ,39.1)	6.2 (-1.1 ,14.1)
Model 2+Employment	1850	35.8 (13.4 ,62.5)	36.3 (8.1 ,72.0)	18.3 (0.9 ,38.7)	6.2 (-1.1 ,14.1)
Model 2+Income[e]	1850	36.0 (13.6 ,62.7)	36.8 (8.5 ,72.4)	18.3 (1.0 ,38.6)	5.7 (-1.6 ,13.5)
Model 2+Pet at home	1850	36.5 (14.0 ,63.5)	36.9 (8.6 ,72.8)	18.7 (1.2 ,39.1)	6.1 (-1.2 ,13.9)
Model 2 (no moving history)	1762	36.0 (13.1 ,63.6)	37.9 (8.8 ,75.0)	16.1 (-1.5 ,36.9)	5.6 (-1.9 ,13.7)

[a]Increment difference of each exposure variables for the health effect estimates are 30 ug/m^3 for $PM_{2.5}$, 20 ug/m^3 for NO_2, 15 ug/m^3 for O_3, and 50% decrease for road distance;
[b]Model adjusted for age, gender, BMI, smoking status, smoking years, cigarettes per day, alcohol consumptions, education, exercise, urbanization, regions, distance to hospital, local Beijing (yes/no);
[c]Cookng includes self-cooking (yes/no), dinner at home (yes/no), and cooking stove (electricity/gas);
[d]Ventilation includes use of fans for cooking (yes/no) and frequency;
[e]Income per month is categorized by (<450, 450-750, 750-1200, 1200-2250,2250-3000, >3000 US dollars)

© 2019 Wang M et al. *JAMA Network Open.*

eTable 4 Effect Modifications in Associations of CAC With Long-term Exposure to $PM_{2.5}$ and NO_2 for Selected Demographics and Risk Factors

Percent Change in CAC (95% CI)[a]	$PM_{2.5}$ (μg/m^3)[b]	P-value	NO_2 (μg/m^3)[b]	P-value
**Gender**		0.01		<0.01
Female	17.6 (2.6, 34.8)		17.9 (1.4 ,37.2)	
Male	42.2 (24.3, 62.7)		45.7 (25.3 ,69.5)	
**Age**		<0.01		<0.01
< 60	17.1 (3.0, 33.2)		21.0 (4.6 ,40.0)	
≥ 60	50.1 (28.8, 75.0)		55.5 (31.8 ,83.6)	
**BMI**		0.22		0.23
< 30	31.3 (17.0, 47.4)		32.7 (15.9 ,51.8)	
≥ 30	10.3 (-16.6, 45.9)		14.4 (-11.9 ,48.5)	
**Smoking status**		0.46		0.45
Never, Former	26.7 (12.2, 43.2)		30.2 (13.1 ,49.8)	
Current	34.6 (14.8, 57.8)		33.8 (12.6 ,58.8)	
**Diabetes mellitus**		0.01		0.09
No	27.0 (10.8, 45.5)		16.7 (8.0, 26.1)	
Yes	62.2 (30.9, 101.0)		31.2 (13.9, 51.0)	
**Regions**		0.38		0.02
North	31.5 (15.5, 49.6)		42.0 (21.7 ,65.6)	
Southeast	15.1 (-10.4, 47.8)		-9.2 (-32.1 ,21.4)	
Southwest	72.3 (-2.7, 205.2)		48.9 (-3.5 ,129.7)	
**Urbanization**		0.20		0.82
Urban	22.8 (6.6, 41.3)		30.0 (10.9 ,52.3)	
Rural	37.5 (18.9, 59.0)		33.1 (10.5 ,60.2)	
**Statin**		0.74		0.86
No	17.1 (8.2, 26.8)		30.5 (13.6 ,50.0)	
Yes	2.3 (0.8, 3.7)		28.7 (7.5 ,54.0)	

[a]Model adjusted for age, gender, BMI, smoking status, smoking years, cigarettes per day, alcohol consumptions, education, exercise, urbanization, regions, distance to hospital, local Beijing (yes/no);
[b]Increment difference of each exposure variables for the health effect estimates are 30 ug/m^3 for $PM_{2.5}$, 20 ug/m^3 for NO_2;

© 2019 Wang M et al. *JAMA Network Open.*

eTable 5 Comparisons of Health Effect Estimates in and Outside Beijing

%Change in CAC (95%CI)[a]	In Beijing (n=2856)	Outside Beijing (n=5312)	p-value
$PM_{2.5}$	10.3 (1.8 ,19.4)	16.1 (9.4 ,23.1)	0.06
NO_2	9.2 (0.9 ,18.3)	14 (7.1 ,21.4)	0.25
O_3	-3.2 (-11.5 ,6)	9.9 (3.5 ,16.7)	0.43

[a]Model adjusted for age, gender, BMI, smoking status, smoking years, cigarettes per day, alcohol consumptions, education, exercise, urbanization, regions, distance to hospital; Health effect estimates were assessed by a SD increase of exposure (Table S4) in or outside Beijing.

© 2019 Wang M et al. *JAMA Network Open.*

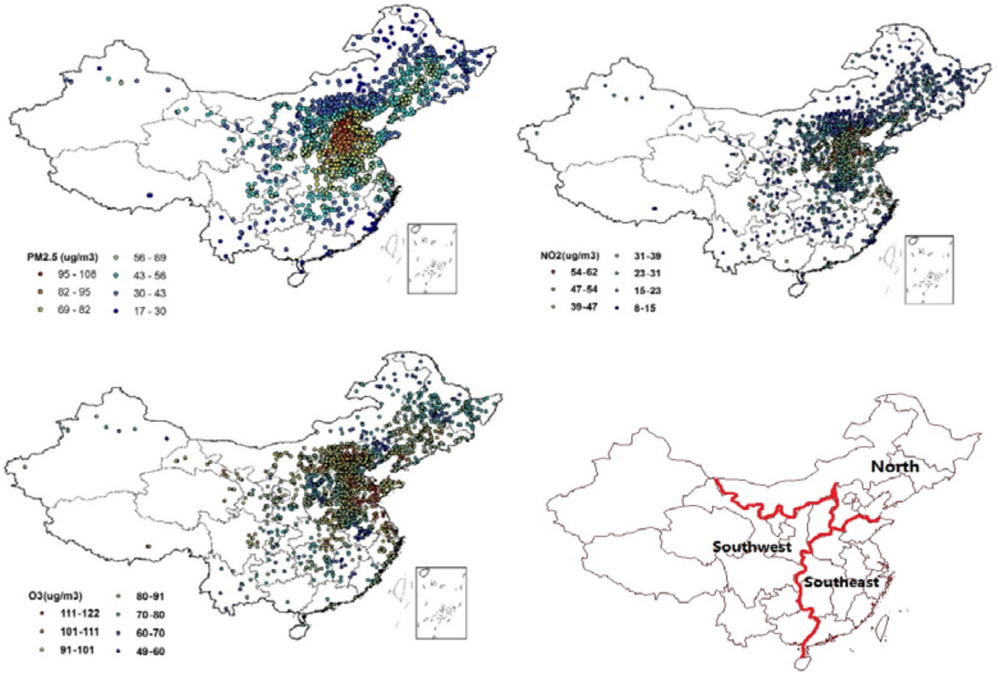

eFigure 1 Spatial Distribution of Estimated Annual Mean Exposure to $PM_{2.5}$, NO_2, and O_3 in the Year of 2015 at the Participants' Home and Regional Classifications.

© 2019 Wang M et al. *JAMA Network Open.*

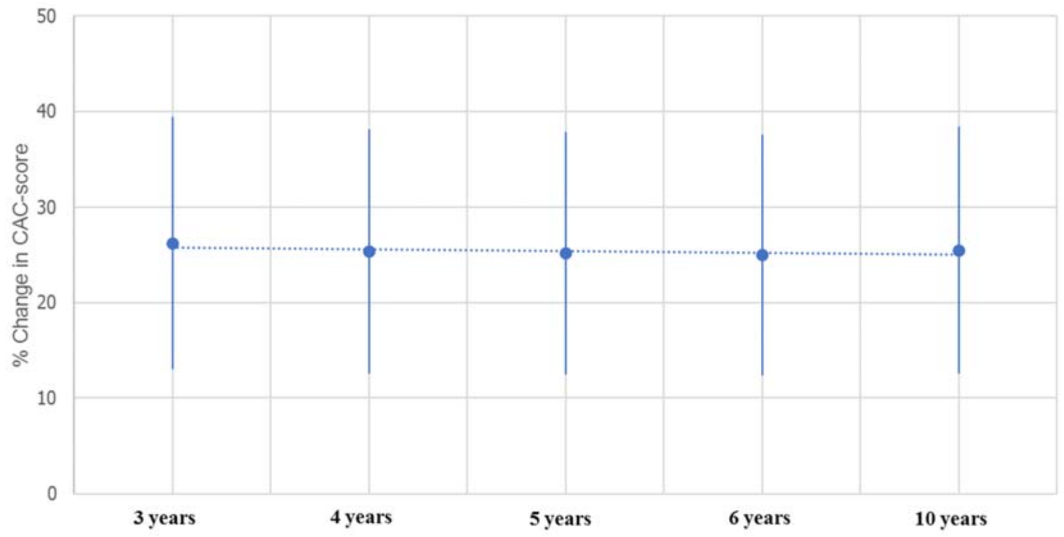

eFigure 2 Percent Change and 95% CI in CAC Associated With $PM_{2.5}$ (30 μg/m^3) Averaged Over Multiple Years Prior to Baseline Using Land Use Regression Model With Adjustment for Satellite-derived $PM_{2.5}$ Observations. Detailed Information on Exposure Estimation for $PM_{2.5}$ Is Described in eMethod: Long-term $PM_{2.5}$ Predictions.

© 2019 Wang M et al. *JAMA Network Open.*

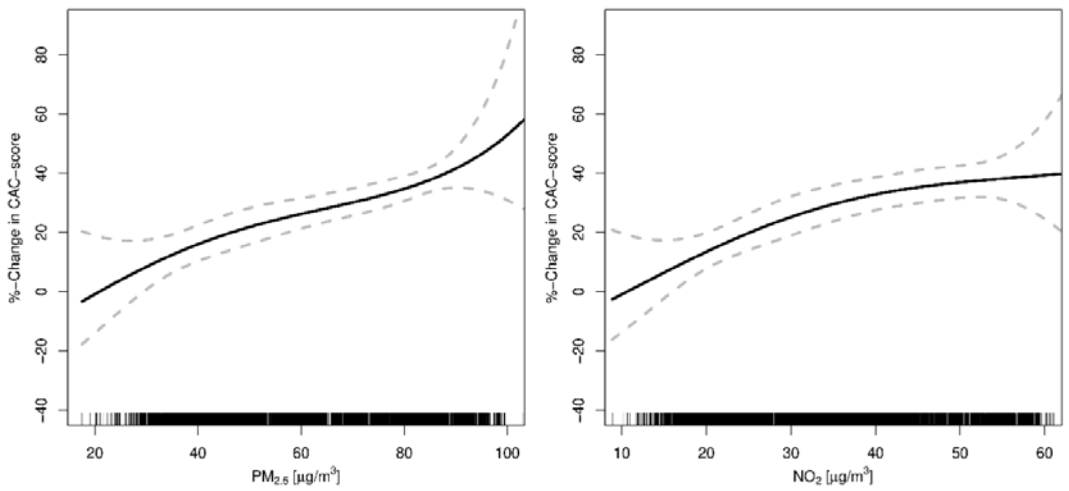

eFigure 3 Concentration-Response Relationships Between Long-term Exposure to $PM_{2.5}$ and NO_2 and the Estimated Percent Change in CAC Score, Modeled by a Natural Spline With 3 Degrees of Freedom.

© 2019 Wang M et al. *JAMA Network Open.*

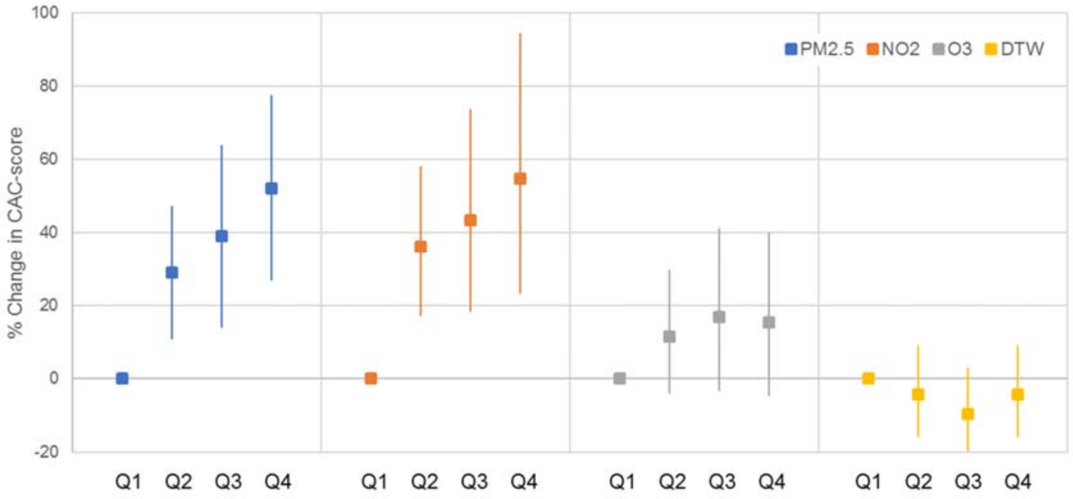

eFigure 4 Associations Between CAC and Long-term Exposure Variables Categorized by Quartiles

(p-Values for Trends of Categorical Exposure Are <0.001 for $PM_{2.5}$ and NO_2, 0.382 for O_3 and 0.237 for DTW). DTW: Distance to roadway. Q1 is the reference exposure.

© 2019 Wang M et al. *JAMA Network Open.*

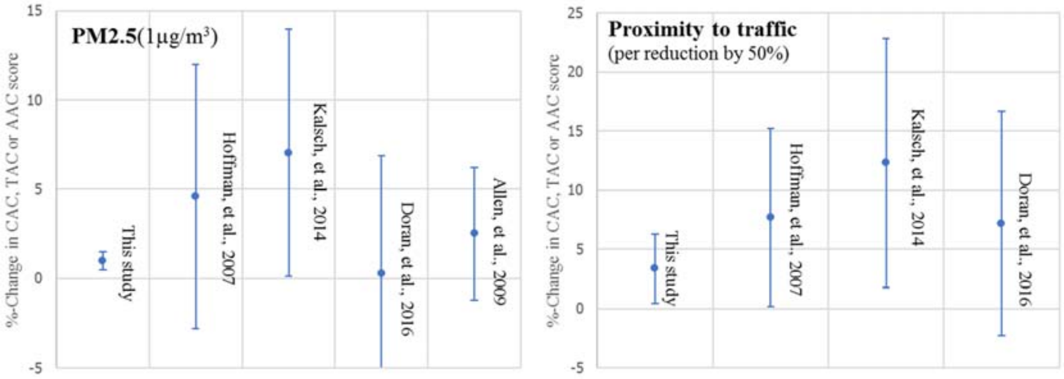

eFigure 5 Comparisons of Heath Effect Estimates for Associations of $PM_{2.5}$ and Proximity to Traffic Exposures With Degrees of CAC, Thoracic Aortic Calcification (TAC) or Abdominal Aortic Calcification (AAC) Between This Study and the Previous Studies[3-6].

© 2019 Wang M et al. *JAMA Network Open.*

eReferences

1. Hou ZH, Lu B, Gao Y, et al. Prognostic value of coronary CT angiography and calcium score for major adverse cardiac events in outpatients. *JACC Cardiovascular imaging.* 2012;5(10):990-999.
2. Ma Z, Hu X, Sayer AM, et al. Satellite-Based Spatiotemporal Trends in PM2.5 Concentrations: China, 2004-2013. *Environmental health perspectives.* 2016;124(2):184-192.
3. Hoffmann B, Moebus S, Mohlenkamp S, et al. Residential exposure to traffic is associated with coronary atherosclerosis. *Circulation.* 2007;116(5):489-496.
4. Kalsch H, Hennig F, Moebus S, et al. Are air pollution and traffic noise independently associated with atherosclerosis: the Heinz Nixdorf Recall Study. *European heart journal.* 2014;35(13):853-860.
5. Dorans KS, Wilker EH, Li W, et al. Residential Proximity to Major Roads, Exposure to Fine Particulate Matter, and Coronary Artery Calcium: The Framingham Heart Study. *Arteriosclerosis, thrombosis, and vascular biology.* 2016;36(8):1679-1685.
6. Allen RW, Criqui MH, Diez Roux AV, et al. Fine particulate matter air pollution, proximity to traffic, and aortic atherosclerosis. *Epidemiology.* 2009;20(2):254-264.

© 2019 Wang M et al. *JAMA Network Open.*
